# Fly Ash as a Potential Adsorbent for Removing Radionuclides from Aqueous Solutions in an Adsorption-Membrane Assisted Process Compared to Batch Adsorption

**DOI:** 10.3390/membranes13060572

**Published:** 2023-05-31

**Authors:** Leon Fuks, Agnieszka Miśkiewicz, Irena Herdzik-Koniecko, Grażyna Zakrzewska-Kołtuniewicz

**Affiliations:** Centre for Radiochemistry and Nuclear Chemistry, Institute of Nuclear Chemistry and Technology, Dorodna 16, 03-195 Warszawa, Poland; i.herdzik@ichtj.waw.pl (I.H.-K.); g.zakrzewska@ichtj.waw.pl (G.Z.-K.)

**Keywords:** radionuclides, coal fly ash, adsorption, membranes, contaminated water, decontamination, liquid radioactive waste

## Abstract

The paper deals with checking the possibility of using fly ash (FA) as a sorbent in the batch adsorption method of removing radionuclides from aqueous solutions. An adsorption-membrane filtration (AMF) hybrid process with a polyether sulfone ultrafiltration membrane with a pore size of 0.22 μm was also tested as an alternative to the commonly used column-mode technology. In the AMF method, metal ions are bound by the water-insoluble species prior to the membrane filtration of the purified water. Thanks to the easy separation of the metal-loaded sorbent, it is possible to improve water purification parameters using compact installations and reduce operating costs. This work evaluated the influence of such parameters on cationic radionuclide removal efficiency (EM): initial pH and composition of the solution, contact time of the phases, and the FA doses. A method for removing radionuclides, ordinarily present in an anionic form (e.g., TcO_4_^−^), from water, has also been presented. The results show, that both batch adsorption of radionuclides and adsorption-membrane filtration (AMF) using the FA as an adsorbent can be effectively used for water purification and in the form of a solid directed to long-term storage.

## 1. Introduction

Water pollution by impurities, such as heavy metal ions, dyes, or organic pollutants, is increasingly becoming a problem with the progress of industrialization. However, the most significant emotions in society are caused by radioactive pollutants, the release of which into the environment must be constantly controlled wherever their sources may appear. This type of water pollution can significantly affect the health of both people and animals, which is why accidents such as Chernobyl or the Fukushima Daiichi nuclear power plant aroused great public interest. The need to quickly deal with the consequences of nuclear accidents results in increased work on developing effective methods for cleaning contaminated land and water. The latter occurs when undesirable radioactive substances enter water streams: surface or underground, oceans, and seas, rivers, and lakes.

Apart from the radioactive incidents, the naturally occurring radioactive material (i.e., material which may contain any of the primordial radionuclides or radioactive elements appearing in the Earth’s crust, NORM) and the anthropogenic radionuclides (i.e., the technologically enhanced naturally occurring radioactive material, TENORM), may be found in water. They come primarily from:

✓ Coal mining and coal combustion residuals,
✓ Hard rock metal, rare earth uranium, or copper mining and production wastes,
✓ Oil and gas mining,
✓ Production of fertilizers.

It is also under discussion whether the above list should also include:

✓ Military explosions and nuclear accidents,
✓ Nuclear fuel reprocessing.

At least a certain amount of radioactive wastewater is also created in nuclear facilities during their normal operation (spent fuel water pools, cooling systems in some types of reactors), in nuclear medicine, and scientific laboratories.

So, the list of radionuclides that may be found in water is long, and the combination of different elements is unspecified.

The literature shows several technologies in use for lowering the concentration of radionuclides in water. They include, above all, chemical precipitation, adsorption, ion exchange, and different pressure-driven membrane processes. Among them, adsorption is one of the preferentially used for water decontamination since it is cheap, easy to operate, and many sorbents are available. By properly choosing the sorbent, adsorption offers high removal efficiency and low operation cost. In many cases, the sorbents may also be regenerated, and the contaminating agent may be obtained in a concentrated form of small volume, so easy to be stored.

Furthermore, the adsorption-membrane filtration (AMF) hybrid process, which combines sorption of the radionuclides with membrane microfiltration, ultrafiltration, or reverse osmosis, seems to be an attractive alternative to the commonly used column-mode technology. This hybrid procedure is accounted among the most prospective methods proposed for the treatment of contaminated water with heavy and/or radioactive metals due to such advantages as [1,2]:

✓ High selectivity is achieved by proper selection of the sorbent,
✓ Ability to separate metal ions even from very diluted solutions,
✓ Relatively high speed of the purification process,
✓ Low energy demand,
✓ The relatively small size of the installation,
✓ The simplicity of the operation,
✓ Small operation costs as compared with other purification methods.

In the method mentioned above, metal ions are bound by the water-insoluble species (as a rule, the macromolecular specimens) prior to the filtration of the purified water. The formed sorbent-metal complexes can be removed using a wide range of membranes. Thanks to this, it is possible to improve water purification parameters, easier separation of sorbent particles loaded with radionuclides from the permeate and reduce operating costs. Moreover, the hybrid method exhibits higher efficiency and lower operational costs than the sorption method in the fixed beds, preferentially used for water purification [3,4,5].

Although fly ash is not evidently a material that may be used as a sorbent of the toxic heavy metals present in aqueous solutions, the bibliography of its use forms quite an extensive collection (see Scopus database, e.g., [6,7,8,9,10,11]). Zeolites and geopolymers, materials traditionally used as sorbents, are also often obtained from the FY. In contrast, the use of FY as a sorbent of radioactive metals is proposed to a much smaller extent. Furthermore, this massive by-product of a thermal power station material is also known as an excellent additive to cement [12]. Since long-term storage of the radioactive wastes is carried out in barrels filled with cement mortars, searching for additives that strongly bind the radionuclides is essential.

These purposes motivated us to test the possibility of using locally available FY as a sorbent of radionuclides from aqueous solutions.

So, the principal goal of the presented work was to examine the possibility of using fly ash (FA) as a potential sorbent that is low-cost and available in extremely great quantities for the removal of trace amounts of radionuclides from water or aqueous solutions. Volatile FA is an industrial by-product formed in power plants and thermal power plants by the combustion of coal, biomass, or a mixture of both. This waste, commonly deposited in landfills, may cause the dangerous pollution of water, air, and soil because of their content of heavy metals (e.g., mercury, cadmium, and arsenic). Fly ash is widely used, e.g., in construction, but due to its similar chemical composition to zeolites, it is also an excellent starting material for synthesizing different porous materials [13]. Among other applications, it was successfully used as an adsorbent to remove different organic contaminants from gases and liquids [14,15,16,17,18].

The paper presents the results concerning the efficiency of the batch adsorption water purification depending on its pH and the presence of certain additives, the effects of the mass of the sorbent used, and the impact of the contact time of the phases. Then, the results of the examination on the possibility of using the adsorption-membrane filtration process [19] are presented.

## 2. Experimental

### 2.1. Materials

Fly ash (FA) from a fluidized bed furnace operating in a combined heat and power (CHP) coal-fired plant (courtesy of the CHP plant Warszawa-Zeran, Poland) was used as received. Sieve analysis showed that about 98.4% of the FA grains were between 0.20 mm and 0.03 mm in diameter. The material was dried in an oven at 70 °C to a constant mass and kept in a desiccator until it was used.

The radiochemical purity of the material was checked by gamma-ray spectrometry before the experiments began. A 120-h test showed no radionuclides except trace amounts of K-40.

All chemicals applied for the experiments were purchased from Sigma-Aldrich Inc. (Szelągowska, Poland) and used as delivered.

The water used was deionized in a Hydrolab HLP 10 UV set (Hydrolab, Wislina, Poland), and the conductivity was 18 MΩ·cm.

Solutions of the carrier-free radionuclides (i.e., which do not contain intentionally added stable isotopes of the same elements, usually in the concentrations of 10−6–10−7 mol/L) of cesium-137 (t_1/2_ = 30.07 y; E_γ_ = 661.7 keV), strontium-85 (t_1/2_ = 64.8 d; E_γ_ = 514 keV), cobalt-60 (t_1/2_ = 5.27 y; E_γ_ = 1.1732 and 1.3325 MeV), and americium-241 (t_1/2_ = 432.2 y; E_γ_ = 59.5 keV) were supplied by POLATOM (Swierk, Poland).

Sodium pertechnetate, Na^99m^TcO_4_, was eluted by 0.9% saline solution from a commercial Poltechnet ^99^Mo/^99m^Tc generator (POLATOM) as ca. 100 MBq/mL solution (The unit of one becquerel (1 Bq) relates to the radioactivity in which one nucleus decays in one second. This is a small quantity of radioactivity so, normally, we use the kilo- and megabecquerel units.).

Aqueous solutions of the desired concentration of the radionuclides were prepared by gravimetric dilution of these radionuclide standards with 10^−3^ M nitric acid prior to checking their purity by gamma spectrometry.

### 2.2. Adsorption of the Radionuclides

Sorption tests followed the spiking of the working solution with the radionuclides forming the carrier-free concentration and adjusting it to the desired pH. The batch adsorption method was used to test FA as a potential sorbent for water purification. Unless otherwise stated, 50 mg of the fly ash placed in the polyethylene test tubes were mixed at room temperature (20° ± 2 °C) with 5 mL of the radioactive solutions by shaking for the required time. The Multi Reax tube shaker (Heidolph Instruments GmbH & Co. KG, Schwabach, Germany) was used throughout the work. Except for the studies of the effect of the equilibration time, the typical contact time was 3 h. In the following, the suspensions were separated by centrifuging (10 min; 14,000× *g* rpm), and the aqueous phases were withdrawn for measurement of the specific concentration (C) of the radionuclides. The initial concentration of the radionuclides in water (*C*_0_) was simultaneously measured. Four radionuclides used were measured at the same time using a PerkinElmer 2480 Wizard2^@^ Automatic Gamma Counter ((Waltham, MA, USA).).

All sorption experiments were performed in triplicate, and the radioactivity concentrations of the solutions were calculated as mean from at least three samples withdrawn from the supernatant liquid and measured five times.

The removal efficiency (*E*_M_; %) of the radionuclides was calculated using Equation (1):

(1) $$E_{M}=\frac{\left(C_{0}-C_{\mathrm{equilibrium}}\right)}{C_{0}}\cdot 100\%$$

where, the specific concentrations of the respective radionuclides *C*_equilibrium_ and *C*_0_ are expressed in Bq/mL^3^.

Each result of adsorption efficiency (*E*_M_) depends on the uncertainty of the main measurement operations, i.e., the radioactivity concentration measurements, the sorbent weighing, and the liquid pipetting. Weighing and pipetting operations, as affected by the relatively small experimental errors (not exceeding 0.01%), were considered non-important in the total measurement uncertainty budget and omitted. Therefore, the combined standard uncertainty of *E*_M_ was calculated using only the values of the standard uncertainty of radiation activity measurements. In the present work, these values (with some exceptions) were 0.5–1.5%.

In a separate experiment, we determined the pH of water equilibrated with the FA. It was found that 3 h equilibration increased this value from 5.0 to 12.5. Thus, purified water has only a technical degree of purity.

### 2.3. Laboratory Ultrafiltration Kit: Adsorption-Membrane Filtration (AMF) Studies

**Figure 1:**
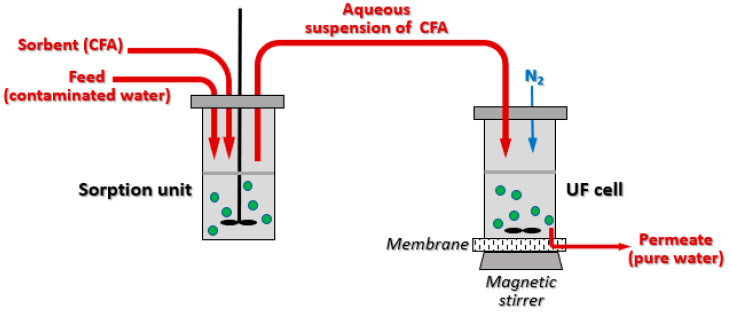
Laboratory kit for the sorption-assisted ultrafiltration.

The sorbent-assisted ultrafiltration experiments on the laboratory scale were carried out with the equipment shown in Figure 1.

The ultrafiltration chamber used in the experiments was a magnetically stirred membrane cell AMICON 8400 pressure-driven filtration compartment that permits studying a broad range of process volumes (up to 400 mL). In the experiments, a polyether sulfone ultrafiltration membrane 

(C_6_H_4_-4-C(CH_3_)_2_C_6_H_4_-4-OC_6_H_4_-4-SO_2_C_6_H_4_-4-O]_n_ was used; with a pore size of 0.22 μm. The diameter of the membrane was 7.6 cm, and the surface in contact with liquid—was 41.8·cm^2^. Both the ultrafiltration chamber and the membranes have been purchased from Merck Millipore (Merck Ltd., Poland). Compressed nitrogen was used as a pressure source to force a constant water flow through the membrane.

During the studies, an initial 120–150 mL feed was poured into the feed tank and stirred for 60 min with the required amount of the FA. Then, the suspension was quickly transferred to the ultrafiltration chamber, and the permeate was collected as the 10 mL fractions, which were then analyzed for the specific radioactivity concentration of the radionuclides using a PerkinElmer 2480 Wizard2^@^ Automatic Gamma Counter.

### 2.4. Methods Used in the Work and the Relevant Instrumentation

#### 2.4.1. Characterisation of the FA

##### Surface Characterization of the FA

Scanning electron microscopy (SEM) analysis is a leading method that delivers information about a solid’s surface and shape. The SEM studies of the dried FA samples were performed at room temperature using a DSM 942 Scanning Electron Microscope (Carl Zeiss Microscopy GmbH, Jena, Germany) after coating the samples with a fine layer of gold. Fractures of the surface of the samples were examined with magnifications of 100, 1000, 3000, and 5000.

When the scanning electron microscope is equipped with an energy-dispersive X-ray microanalyzer (EDX), it is possible to correlate the morphological characteristics of the solid and to obtain a mapping of the elemental concentration profiles. The elemental composition analysis in the micro-areas was carried out with the microanalysis system Quantax 400 (Bruker, Leipzig, Germany). Different options for the signal collection were used and analysed for an area of about 0.01 mm^2^.

##### Electrical Potential of the Interface between Bulk Water and a Solvent Attached to the Surface of a Sorbent (Zeta-Potential)

Zeta potential (ζ_n_) is an electrical potential that characterizes the interface separating a mobile fluid from a fluid attached to the surface of a solid. Thus, the determined value of zeta potential allows us to predict the possibility of sorbate binding by the sorbent particles.

Zeta potential was determined by measuring the velocity of movement of the CFA particles forced by the electrophoresis. Their movement speed is properly related to the applied electric field strength and their zeta potential. A Zetasizer Nano ZS instrument, Malvern, UK, was used to determine the electrophoretic velocity, and the dynamic light scattering method was applied.

##### Thermal Analysis

Thermal gravimetric analysis (TG) is a technique applied to determine the temperature at which physical or chemical changes happen upon heating or cooling the analyzed substance. This technique is among the most widely applied analytical methods used to characterize adsorbents. Derivative thermal gravimetric (DTG), in turn, offers information on the decomposition of the sample at a specific temperature through analysis of the peaks. For example, one may distinguish between the process’s endo- and exothermal character.

Thermogravimetric (TGA) and difference thermogravimetric (DTG) analyses were made in the flowing air (flow rate: 100 mL·min^−1^) by heating with a temperature increase rate of 10 °C·min^−1^. Scanning temperatures ranged from 40 to 900 °C. Linseis Stapt 1600 (Linseis Corporation, Selb, Germany) analyser was used.

#### 2.4.2. Radioanalytical Methods

##### Radiometric Analyses of the Initial- and Equilibrium Aqueous Solutions/Fractions Collected in the AMF

Four radionuclides used in the experiments were measured simultaneously in the 1 mL samples using a PerkinElmer 2480 Wizard2^@^ Automatic Gamma Counter. Each sample was measured five times, then a mean value and the standard deviation were calculated.

##### Checking the Purity of the Radionuclides

The purity of each radionuclide was checked in the 5 mL sample using the gamma-ray spectrometer with HPGe semiconductor detector (CANBERRA, ORTEC, Oak Ridge, TN, USA) with an efficiency of 45%. Every spectrum was collected for 1 h.

##### Production of the Saline Aqueous Solutions Containing Tc-99m

The Mo-99/Tc-99m medical generator (GE Healthcare, supplied by Biker, Warsaw, Poland) was equipped with a chromatographic column filled with an Al_2_O_3_ bead loaded with the parent radionuclide Mo-99 in the form of the sodium molybdate (Na_2_MoO_4_). When the radionuclide decays into the pertechnetate, NaTcO_4_, the latter was eluted with the 0.9% NaCl solution prior to each experiment. The radiochemical purity of the eluate was checked using gamma-ray spectrometry.

#### 2.4.3. Supporting Analytical Methods

Checking the chemical composition of aqueous solutions containing a mixture of salts was performed by ion chromatography using the chromatograph DIONEX ICS-5000 DC (DIONEX, Sunnyvale, CA, USA).

Analysis of metal content in the FA was conducted using laser ablation inductively coupled plasma mass spectrometry (LA-ICP-MS). The ELAN DRC II inductively coupled plasma quadrupole mass spectrometer (Perkin-Elmer) was used.

## 3. Results and Discussion

### 3.1. General Characterization of the Fly Ash (FA)

Inspection of the surface of the studied solid material for its elemental composition was performed by the energy-dispersive X-ray spectroscopy (abbreviated as EDS, EDX, or EDXA) method. Parameters characterizing the fly ash (FA) and its chemical composition are shown in Table 1.

As can be seen from Table 1, the composition of metal oxides of the fly ash used in this study remains in good agreement with the literature data, e.g., with [20]. The FA elemental composition, as is shown in Table 1, also is in good agreement with the composition of ash obtained from the combustion of coal dug in Polish mines [21]. The classification system of fly ash materials is based on their chemical composition, i.e., on the mass ratios obtained for the SiO_2_, Al_2_O_3_, Fe_2_O_3_, MnO, Na_2_O, K_2_O, MgO, CaO, TiO_2_, P_2_O_5_, and SO_3_ components, the composition of the studied material classifies it into the ferrocalsialic group [22]. Furthermore, as the summary concentration of SiO_2_, Al_2_O_3,_ and Fe_2_O_3_ is greater than 50% of the fly ash, and CaO content is smaller than 18%, according to the ASTM C618 requirements, the material may be included in class F of the fly ash group [23].

### 3.2. Characterization of the FA Surface by Scanning Electron Microscopy (SEM)

**Figure 2:**
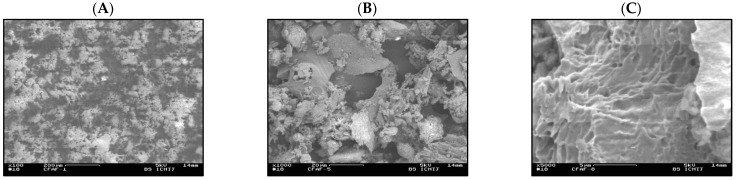
SEM micrographs of the fly ash (FA) from the Warszawa-Zeran heat and power plant. Enlargements: (**A**) 100 times, (**B**) 1000 times, (**C**) 5000 times.

Surface characterization of the solid by the SEM method offers topographical information about the material and allows for the comparison of different parts of the specimen simultaneously. The micrographs of the fly ash, tested in the present work as a potential sorbent of the radionuclides, acquired for different enlargements, are shown in Figure 2.

As can be seen from Figure 2A,B, some grains seen by the microscope have an almost spherical shape. Such particles are favourable for usage as the filler in cement mixtures, often used in radioactive waste storage [24]. However, one can also detect some clusters of a different, irregular shape. As shown in Figure 2C, no evident macro- and microchannels can be seen, which may enhance the adsorption of metal ions.

### 3.3. Thermogravimetric Analysis of the Coal Fly Ash (FA)

**Figure 3:**
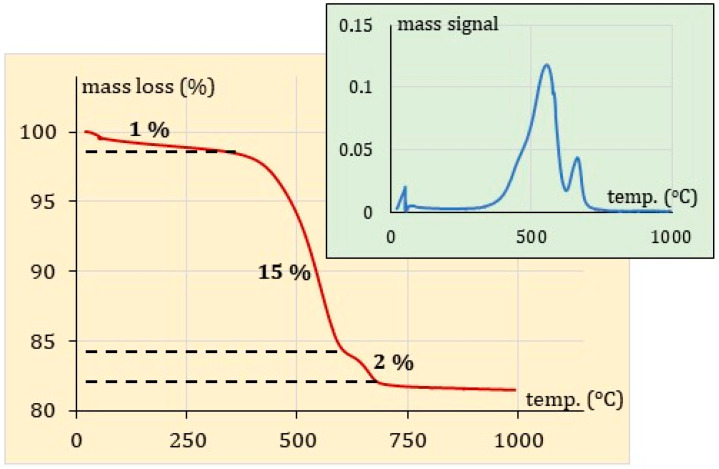
Thermogravimetric analysis TGA and DTG of fly ash.

A thermogravimetric plot of the FA is indicated in Figure 3. The total mass loss is not great and does not exceed 20%. Such a value corresponds well to the thermal mass loss observed, e.g., by Marruzzo et al. [25]. This means that it is not likely that the mass of a concentrate containing radionuclides can be significantly reduced in preparing its landfillable form (e.g., by cementation).

Figure 3 shows three mass loss temperature ranges with the well-separated maxima of the DTG curve. The first mass decrease (of about 1% that appears below 100 °C), may be attributed to the moisture removal and release of the structural water. This process takes place up to about 350 °C. The main mass loss, of about 15% between 350 and 640 °C, is probably caused by the decomposition of carbonates. The last mass decrease, in turn, of about 2% (at about 650 °C) may be assigned to the combustion of the unburnt carbon [25]. This range of temperatures corresponds well with the value obtained in our previous work on activated carbon (630 °C) [26]. In the temperature range of 600–700 °C, also the thermal decomposition of calcium carbonate (usually present in the FA) may occur [27].

### 3.4. Zeta Potential of the FA Surface

The electrokinetic potential (zeta- or ζ-potential) describes the potential occurring at the surface of a solid (or other dispersed particles, e.g., emulsions) in contact with the slipping plane, i.e., the interface which separates the electrolyte bulk solution from the ions fixed on the surface of the solid phase [28]. So, as the existence of the ζ-potential implies the electrokinetic phenomena, in this work, we may relate its value to the affinity of the sorbent surface towards the ions present in the solution.

Within the presented studies, we have determined values for the FA ζ-potential to be −21.6 and −24.8 mV for the pH = 5 and pH = 8, respectively. Both obtained values correspond well with those reported by Nägele and Schneider [29]. From the literature on the electrochemical properties of matter, it is known that values of ζ-potential being about ±25 mV describe systems that are of a certain degree stable and form colloidal aggregates to a limited extent [30]. So, the values of the ζ-potential determined in our work suggest the usefulness of the FA as a cation sorbent, as its surface is sufficiently developed to allow for the formation of sufficiently strong interaction with metal cations.

Moreover, it has been published already that the surface of the FA particles is positively charged at low pH and has a negative charge at higher. A point of zero charge (PZC) is at a pH of about 2.8 [20]. So, these data allow the assumption that metallic cations will be adsorbed onto the negatively charged fly ash particles only at a pH higher than 2.8.

### 3.5. Batch Sorption of the Cationic Radionuclides

Batch sorption experiments were conducted to study the sorption behavior of the mono-, di- and trivalent cationic radionuclides on the fly ash (FA) obtained from the coal combustion at the Zeran power plant in Warsaw. Sorption studies of the multimetal systems were performed, similarly to our previous research [31]. The monovalent cations were represented by Cs(I)-137, the divalent cations by Sr(II)-85 and Co(II)-60, and the tetravalent cations—by Am(III)-241. The solutions were carrier-free, so one metal did not disturb the sorption of the others.

#### 3.5.1. Effect of Contact Time of the Phases on Adsorption

In designing the adsorption process as the radioactively contaminated water’s purification tool, the optimum time for leading the process should be established. Generally, the adsorption rate initially increases rapidly. Then the constant removal efficiency is reached. Further growth of the contact time does not significantly change the equilibrium concentration of the removed metals. It means that the adsorption process achieved equilibrium.

**Figure 4:**
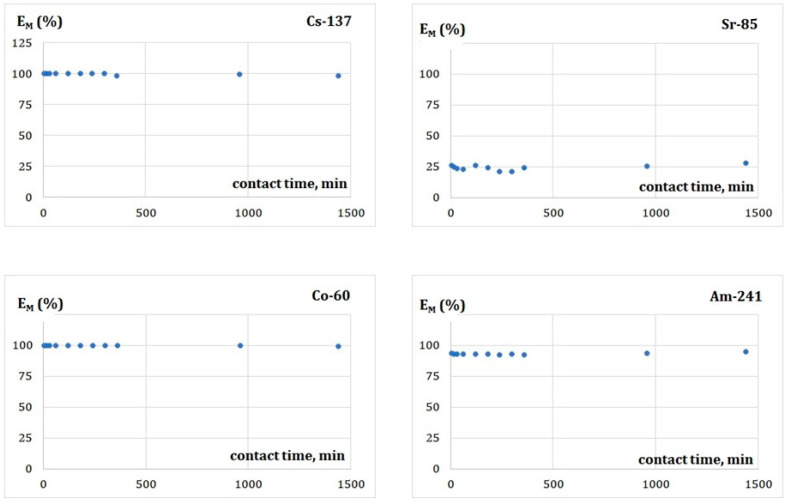
Impact of contact time of the phases on adsorption efficiency.

Figure 4 shows the metal removal efficiency (E_M_; %) concerning the contact time of the phases. The obtained results of the adsorption of Cs(I), Sr(II), Co(II), and Am(III) ions by the FA indicate that with the increase of time, the E_M_ values neither does not increase nor does not decrease. So, the equilibrium was reached relatively quickly for all the investigated systems. Within 5 min–24 h, the E_M_ values are constant at the levels: 99.6 ± 0.7% for Cs(I), 24.4 ± 2.2% for Sr(II), 99.7 ± 0.2% for Co(II), and 93.1 ± 0.7% for Am(III) cations. So, the process of the radionuclides’ removal is quick, and even 5 min. of contacting the phases is sufficient to reach equilibrium. Due to the short time of reaching equilibrium, we have not determined a kinetic model of the process in the presented work. However, A review of the literature on the sorption of non-radioactive metals indicates that the pseudo-second order model describes the sorption process better than the other models [32].

Sorption removal of Cs(I), Sr(II), and Co(II) metal ions from aqueous multimetal solutions by different sorbents was examined already, e.g., by Abou-Lilah et al. [33,34]. Similarly to our results, data published in these papers demonstrate that sorption capacity values of these metals follow a pattern: Cs(I)~Co(II) >> Sr(II). Such a line does not correlate with a pattern of the ionic potentials (charge-to-ionic radius ratios) that is proportional to the strength of an ionic bond. The observed peculiar values of the E_M_ found in our work for Sr(II) we referred to the Sr(II) speciation, which is highly dependent on pH and the composition of the purified aqueous solution. However, data available in the literature show that the speciation of all studied metals in the examined pH range is similar, and the dominant form is the M^n+^ ion [35].

So, the explanation for such a peculiar behavior of strontium(II) might lie in the different crystal structures of the oxides forming the FA. Qualitative XRD spectra recorded by McCarthy et al. [36] indicate that low-calcium/Class F fly ash (as our material is) consists mainly of the crystalline phases of quartz (SiO_2_), mullite (Al_4+2x_Si_2−2x_O_10−x_, with x ranging between about 0.2 and 0.9), hematite (Fe_2_O_3_) and magnetite (Fe_3_O_4_) in a matrix of aluminosilicate glass (K_2_O-Na_2_O-Al_2_O_3_-SiO_2_ system). The crystal structure of mullite consists of chains formed by edge-connected AlO_6_ octahedra [37], and hematite (α-Fe_2_O_3_) has a corundum-type crystal structure with the oxide ions O^2−^ forming a hexagonally close-packed array and Fe^3+^ ions occupying the octahedral interstices [38]. Finally, the magnetite structure is an inverse spinel, with O^2−^ ions forming a face-centered cubic lattice and iron cations occupying interstitial sites (space between the atoms’ packing in the crystal structure). Half of the Fe^3+^ cations occupy the tetrahedral sites, while the other half, along with Fe^2+^ cations, occupy the octahedral sites. It seems that the interaction of the square antiprismatic Sr^2+^ ions [39] with the oxide ions present on the surface of the sorbent components is less favorable than this of the large, 12-coordination Cs^+^ ions, octahedral Co^2+^ ions and tricapped trigonal prismatic Am^3+^ cations.

#### 3.5.2. Effect of the Solution pH on Adsorption of the Radionuclides

**Figure 5:**
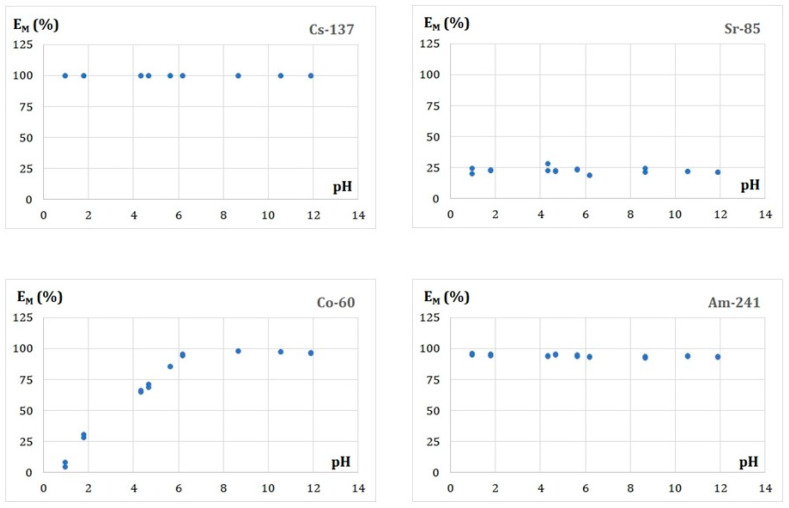
Effect of the solution pH on the radionuclide adsorption efficiency.

The acidity of a purified aqueous solution is also an essential parameter influencing the adsorption process because it affects both an adsorbent’s surface charge and the metal ions’ speciation. The initial acidity of the solutions studied in the present work was prepared within the range of pH 1 to pH 12 by stepwise addition of 10^−2^ M HNO_3_ or 10^−2^ M NaOH. Detailed experimental conditions of the sorption studies were: initial metal concentrations: 10^−6^–10^−7^ mmol/L; the FA doses: 30.1 ± 0.1 g/L; the volume of the solution: 5 mL; temperature: ambient. The obtained E_M_ (%)-pH diagrams are shown in Figure 5.

These results reveal that the adsorption behavior of Cs(I), Sr(II), and Am(III) form a plateau over the entire range of pH values. In detail, the E_M_ values are about 100.0 ± 1.0% for Cs(I), 22.6 ± 2.1% for Sr(II), and 94.3 ± 1.0% for Am(III) metals. These values are consistent within the limits of the experimental error with the values obtained for the dependence of the E_M_ on the contact time of the phases. For Co(II), in turn, one can observe an increase of the E_M_ values from 6% to 96.8 ± 1.2%, for the pH values increasing from 1 to 6. After that, the E_M_ values remain almost constant up to pH 12.

In a separate experiment, we determined the pH of water equilibrated with the FA. It was found that 3 h equilibration increased this value from 5.0 to 12.5. Thus, purified water has only a technical degree of purity.

#### 3.5.3. Effect of the FA Dose on Adsorption

**Figure 6:**
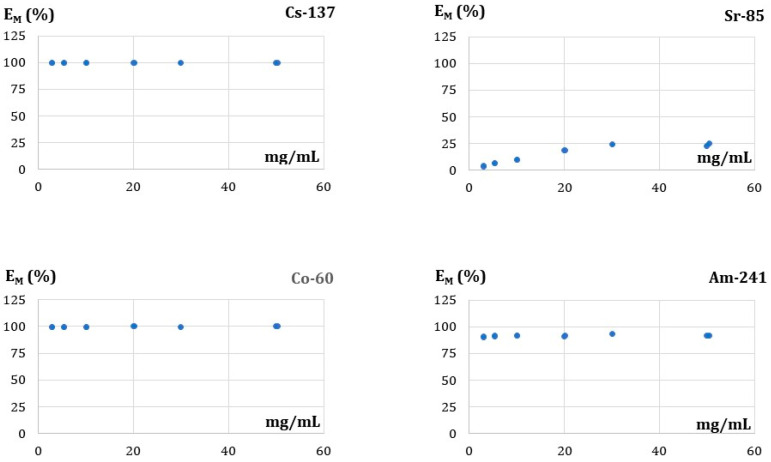
Effect of the FA dose (mg/mL) on radionuclides’ removal efficiency (EM, %).

The optimum dose of the sorbent is accounted for among the key factors because of the adsorption process. In this work, the effect of the fly ash dose on the sorption of the radionuclides was checked in ambient temperature, pH 5–6, contact time—180 min, and the sorbent doses within the range of 3–50 g/L. The initial radionuclide concentration was below 10^−6^ molar. The results are shown in Figure 6.

As can be seen from Figure 6, the constant removal efficiency of 100.0 ± 0.1, 99.7 ± 0.3, and 91.6 ± 0.7 for Cs(I), Co(II), and Am(III), respectively, were observed when the dosage varies between 3 g/L and 50 g/L. For the comparison, constant values of the Cu(II) removal efficiency were observed for the FA dosage greater than 2 g/L while a sharp increase was observed for the dosage in the 0.5–1.5 g/L [40]. This indicates that several the existing active sites, even in the lowest sorbent dosage, is sufficient for the removal of all radionuclides from the solution. In the case of Sr(II), in turn, an increase in removal efficiency was observed when the FA dose grows from 3 g/L to 25 g/L. After that, the removal efficiency remains constant (24.0 ± 1.1) % for the doses from 30 g/L to 50 g/L. Thus, it can be concluded that a dose of about 30 g/L is the optimum value for all the radionuclides studied in the above conditions.

#### 3.5.4. Simulated Liquid Radioactive Waste

The existence of salts in radioactively contaminated water and aqueous solutions affects their many properties in relation to pure water. Despite this, some works have not considered this fact in their studies on the partition equilibria of the separation of different solutes. As a part of the presented studies, we have examined the sorption of the radionuclides by the FA in the presence of a synthetic aqueous mixture of salts, which composition corresponds to the real radioactive waste collected in the Radioactive Waste Management Plant, Poland. In detail, we have prepared a solution containing CsNO_3_ (in a concentration of about 0.6 g/L), NaNO_3_ (2 g/L), KNO_3_ (1.2 g/L), MgCl_2_ (1 g/L), and CaCl_2_ (0.3 g/L). In a separate experiment of shaking an aqueous solution containing salts and the radionuclides, but without the addition of a sorbent, we showed that, even if the radionuclide sorption on the walls of the experimental set of the radionuclides is observed, it is less than 0.01% of the radionuclides.

**Figure 7:**
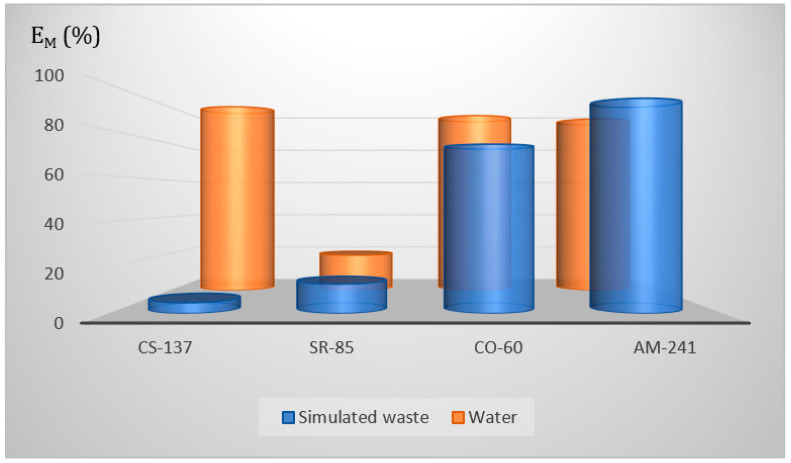
Comparison of the removal efficiency (E_M_) of the radionuclides from water and the solutions containing salts.

Experimentally obtained results of sorption of all four studied radionuclides are presented in Figure 7, both from pure water and the saline solution. A significant decrease in the removal efficiency (E_M_, %) may be observed for the simulated waste as compared to water (both for the pH 5). Additionally, it should be noted that this decrease is much smaller for the multivalent cations as compared with the monovalent cesium. In detail, for Am(III), this decrease is almost unnoticeable (about 2–3%), while for Sr(II) and Co(II), it rises to about 30–40%. In the case of Cs(I), it already has a value of about 20 times. This unusually large drop in E_M_ is probably due to the presence of a significant excess of natural cesium nitrate, effectively competing with the radiocesium for the FA binding sites.

### 3.6. The Adsorption-Membrane Filtration (AMF) Hybrid Process of Removal of the Cationic Radionuclides

Conventional water treatment procedures, such as flotation, chemical precipitation, or adsorption, are not suitable for the removal of metals present in low concentrations. Ion exchange and sorption, with, e.g., activated carbon, processes are to a high degree expensive, especially when dealing with significant volumes of water. In addition, in the case of sorption, the high costs of the process of separating large amounts of sorbent from the aqueous solvent must be considered. The hybrid adsorption-membrane filtration (AMF) process joins the outstanding ability to remove metals from water by sorption with a noticeable capability of the membrane in the filtration. Compared to other membrane processes, ultrafiltration and microfiltration do not need high pressures to be applied. So, in the presented work, we have examined the possibility of using this variant of the membrane processes in combination with the fly ash sorption to remove the mono-, di-, and trivalent radionuclides from aqueous solutions.

#### 3.6.1. Choice of the Membrane

In the presented work, three membrane materials were tested, namely RC 10 KDa (PM UC010, Microdyn-Nadir, GmbH, Wiesbaden, Germany), PVDF 0.1 µm (Maine Manufacturing LLC, Sanford, ME, USA), and PES 0.22 µm (Maine Manufacturing LLC, USA).

**Figure 8:**
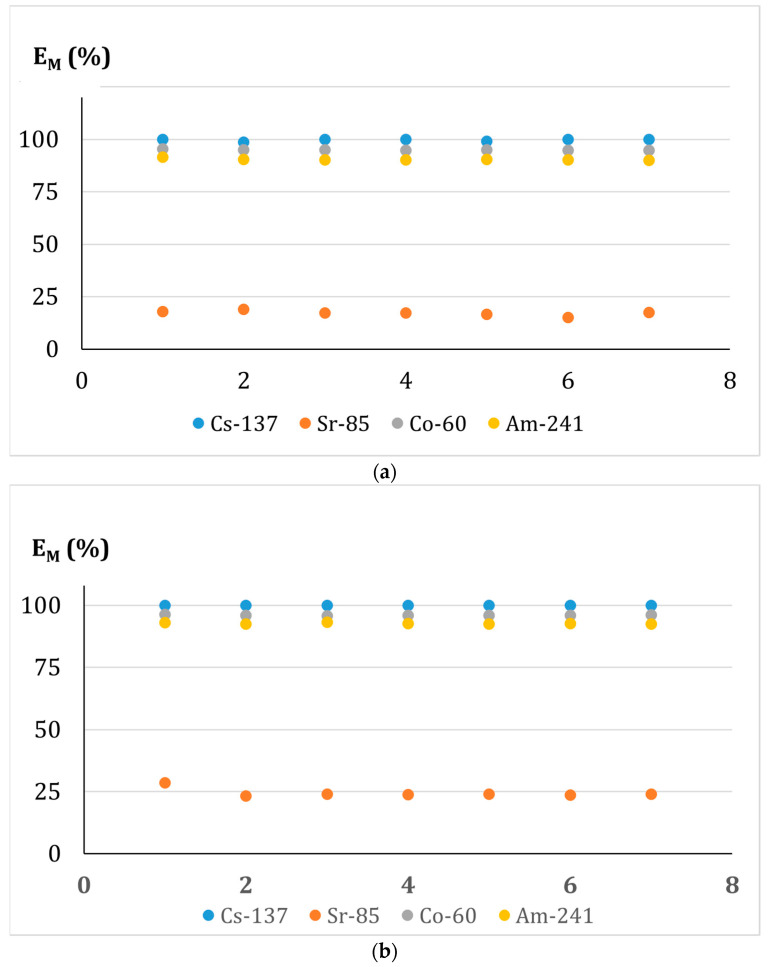

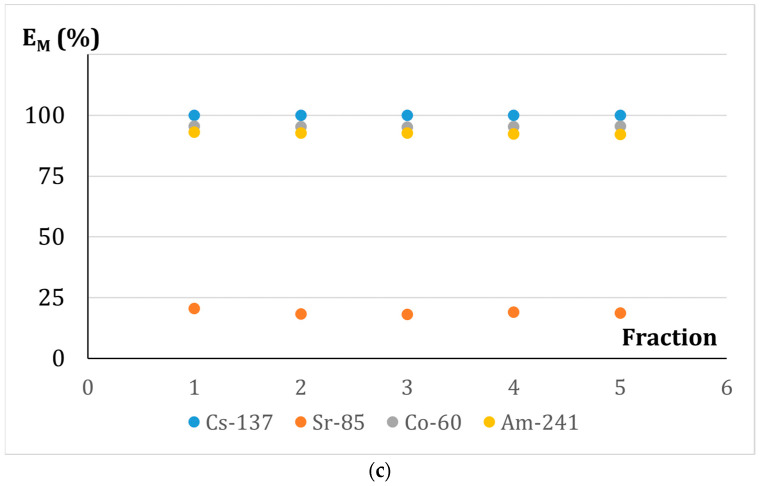
Choice of the membrane suitable for the removal of radionuclides. (**a**) RC 10KD; (**b**), PVDF 0.1 µm; (**c**), PES 0.22 µm.

One gram of the fly ash was introduced into the ultrafiltration cell containing 150 mL of a solution of four radionuclides studied (pH 5). The suspension was magnetically stirred for 1 h before starting filtration of the suspension forced by a nitrogen stream. The permeate was collected as 10 mL fractions, which were confectioned into 1 mL portions and sent for the analyses of the removal efficiency of radionuclides. The determined removal efficiency for all three membranes is presented in Figure 8.

Summarizing the results obtained in this part of the work, it may be stated that each of the membranes tested provides a comparable quality of purification of water from Cs(I)-137, Sr(II)-85, Co(II)-60, and Am(III)-241. So, in the following tests of the adsorption-membrane filtration (AMF) hybrid process, we decided to use a PES membrane with a pore size of 0.22 μm as it has been shown that the use of a membrane with smaller pores does not increase the efficiency of radionuclides removal from the tested solution.

#### 3.6.2. Effect of Mass of the Fly Ash Used as Sorbent in the Adsorption-Membrane Filtration (AMF) Hybrid Process

**Figure 9:**
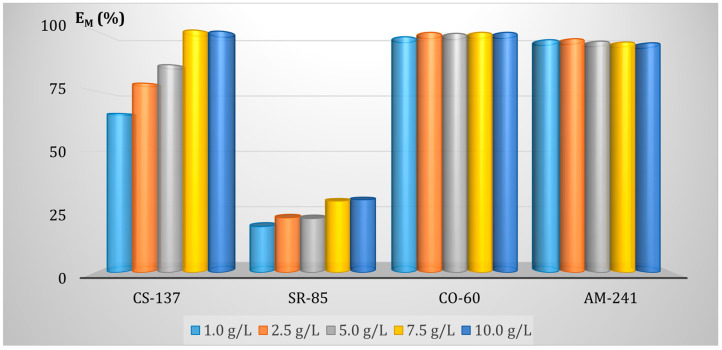
Effect of the fly ash mass on the radionuclides’ removal from aqueous solutions using adsorption-membrane filtration (AMF).

The fly ash dosage’s effect on the radionuclides’ removal efficiency was studied for the adsorbent doses of 1.0, 2.5, 5.0, 7.5, and 10.0 g/L. The pH of each solution was kept at a constant value of 5. Each solution was agitated with the sorbent for 1 h. on the magnetic stirrer. Then the samples were subjected to filtration. The E_M_ values for all sorbent doses and all radionuclides are shown in Figure 9.

As can be seen, for Co(II) and Am(III), even the smallest dose assures the total removal of the radionuclides. In turn, the dosage for Cs(I) and Sr(II) must be at least 7.5 g/L. Thus, to completely remove the radionuclides from water, the sorbent must be added in the amount of at least 7.5 g/L.

It should also be noted that the effectiveness of radionuclide removal by the hybrid sorption-membrane filtration method is comparable to the batch sorption process carried out on a laboratory scale, however in the case of the hybrid process a less amount of sorbent is needed, only 7.5 g/L (compared to batch process- 30 g/L) to achieve the same removal efficiency of all radionuclides (Figure 6). In the case of Sr (II), even a bit higher removal efficiency was achieved in the hybrid AMF process. It can be explained by the higher mass transfer coefficient value obtained in the hybrid AMF process case.

#### 3.6.3. Effect of the Contact Time of the Phases in the Adsorption-Membrane Filtration (AMF) Hybrid Process

**Figure 10:**
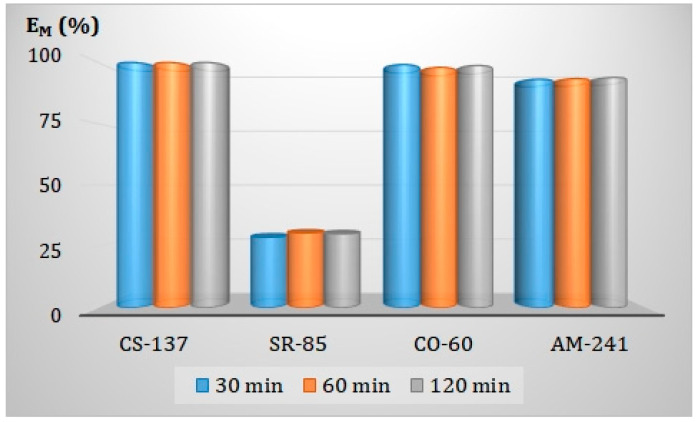
Effect of the phases contact time on the radionuclides removal from water in the adsorption-membrane filtration (AMF).

Since the hybrid AMF process consists of sorption and ultrafiltration processes, it became necessary to check the impact of the contact time of treated water with the sorbent on the effect of the purification. In the presented studies, we contacted both phases for 30 min, 60 min, and 120 min, respectively. The obtained E_M_ values for all the radionuclides studied are shown in Figure 10. It has been found that the phases’ contact time does not significantly affect the radionuclides’ removal from water in the adsorption-membrane filtration (AMF) process. The detailed length of the time necessary to effectively provide the process will be determined during its design.

#### 3.6.4. Membrane Fouling

The water flux (J_w_) was determined experimentally before the AMF process under a transmembrane pressure of 0.5 bar. The measured J_w_ values were in the range of 0.089–0.095 m^3^/(m^2^s). The permeate flux during the AMF process was maintained at 0.083 ± 0.025 m^3^/(m^2^s). As a result of such a high flow rate of permeate, and quite a small volume of the feed solution (150 mL), the filtration process was short enough that the drop in permeate flux was not noticeable over time. Even in an experiment with a larger amount of feed solution (800 mL) using a buffer tank, we did not observe a significant decrease in permeate flux over time.

Nevertheless, membrane fouling will occur during the filtration of much larger volumes of the feed solution due to the deposition of FA particles on the membrane surface. The membrane fouling caused by FA was observed when the removal of phosphate ions (PO_4_-P) from an aqueous solution by means of fly ash in a crossflow microfiltration system was investigated [41]. In our case, the deposit of the FA formed on the membrane surface has been observed at the end of the experiment. It did not noticeably affect the permeate stream. However, removing the membrane surface was very difficult after each experiment. This limited the regeneration of the membrane for its reuse. Applying washing solutions (citric acid and NaOH solution) resulted in only partial removal of sediment from the membrane surface. On the other hand, this layer of FA can be treated as a “secondary membrane”, which can enhance the separation of radionuclides. There are known examples in the literature that membranes made of FA are used to purify liquid aqueous solutions [42].

#### 3.6.5. Simulated Liquid Radioactive Waste

Also, in the examination of the method of water purification using a hybrid sorbent-assisted microfiltration, we decided to check whether the presence of weight amounts of salt affects the efficiency of the process. The salt content in the purified solution was the same as in the batch sorption experiments. All the most important parameters of the experiment were the same as those used in the other AMF experiments.

**Figure 11:**
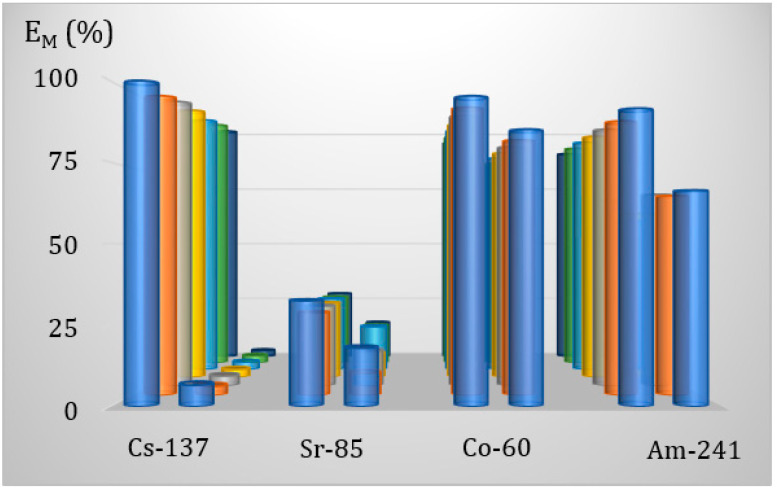
AMF purification of water as compared with salts containing solution. The consecutive fractions of the membrane filtration process are shown in the depth of the graph.

The results presented in Figure 11 reveal that, like in the batch sorption experiments, the coexistence in water of significant amounts of the salts notably reduces the possibility of water purification.

### 3.7. Removal of the Metal Oxo-Anionic Radioactive Contaminants from Water

With the rapid development of nuclear power production and as a result of tests of nuclear weapons, the environment decontamination from the radioactive nuclides such as ^129^I (t_1/2_ 1.7 × 10^7^ years), ^93^Mo (t_1/2_ 4 × 10^3^ years), ^99^Tc (t_1/2_ 2.1 × 10^5^ years), and ^79^Se (t_1/2_ 3.27 × 10^5^ years) which exist in an anionic form, as well as disposal of the radioactive wastes containing these radionuclides, starts to be a key problem. The aforenamed radionuclides weakly attach to most minerals and sorbents and are strongly mobile with different aqueous streams. The sorption of differently charged metal cations has been broadly studied [43]. However, much less data are available for the sorption of the metallic anions.

Technetium exists in nine oxidation states, namely from −1 to +7. The most stable are cationic +4 form, +5, and +7 (both anions). Naturally, technetium exists in the form of the pertechnetate (pertechnetate oxyanion ^99^TcO_4_^−^, chemically being like the permanganate), and to a lesser extent, as a tetravalent product of the pertechnetate reduction [44]. So, if technetium-99 is found in water, it is a problematic pollutant because of its high mobility in the environment [45].

In our previous work, we have already proposed that the batch sorption removal of pertechnetate anions from water may be realized if its reduction to the cationic form was previously performed [46]. Then, the possibility of using the ultrafiltration method supported by sorption (SAUF) has been proven to be an efficient method for purifying water contaminated with the pertechnetates [19]. This study has examined the sorption potential of the FA for Tc-99m as a surrogate for Tc-99. Technetium-99m radionuclide (t_1/2_ 6.01 h; γ-ray emitter of the easy-to-detect radiation energy), was used to facilitate the determination of the radioactivity concentration.

**Figure 12:**
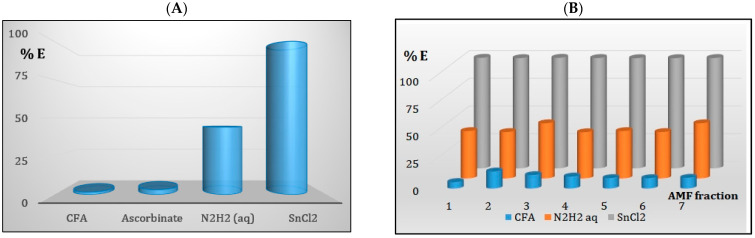
Removal of Tc-99m from wastewater: (**A**) batch sorption, (**B**) AMF procedure.

Due to the negative value of the z-potential of the FA, pertechnetate anion does not bind strongly to the sorbent particle. So, it cannot be removed effectively from aqueous solutions [47]. However, we may expect that the presence of a reducing agent should enhance the Tc-99m removal from water. Below, we present the results of our efforts to remove ^99m^Tc(VII) from aqueous solutions using sorption by the FA. The obtained results are shown in Figure 12.

The left part of Figure 12 presents values of the technetium removal efficiency (RE) from water containing the radionuclide in the natural form of Tc-99m, together with the data obtained in the presence of the stannous chloride (SnCl_2_), ascorbic acid, and the hydrazine hydrate. In detail, these values are 1.67 ± 0.08, 3.24 ± 0.1, 44.01 ± 0.02, 99.91 ± 0.01 percent for the pertechnetate form, and after the addition of the ascorbate, N_2_H_2_, and SnCl_2_, respectively. The presented data indicate that, although the pertechnetate anion does not sorb on the FA particles—after adding a reducing agent used for the laboratory production of radiopharmaceutical kits (SnCl_2_ [48]), it can be completely removed from the water. However, popular green reductors are not as effective in reducing pertechnetates as stannous chloride. Searching for other effective green reductors seems, therefore, recommended.

As can be seen in the right part of Figure 12, using the AMF procedure, water purification efficiency is 10.42 ± 2.93, 44.34 ± 3.73, and 99.54 ± 0.14 percent for the pertechnetate, and after the addition of SnCl_2_ or hydrazine, respectively. These results are completely consistent with batch sorption.

## 4. Conclusions

The obtained results show that both batch adsorption of radionuclides and adsorption-membrane filtration (AMF) using the FA as a sorbent can be effectively used for water purification.

The adsorption process of the radionuclides removal is quick, and even 5 min. of contacting the phases is sufficient to reach equilibrium. In the case of Cs(I), Sr(II), and Am(III) sorption is independent of the pH of the water to be purified. For Co(II), in turn, the optimum condition for sorption is a pH range above 6. For Cs(I), Co(II), and Am(III) we observed that the dosage of 3 g/L is sufficient for their removal from aqueous solutions. For Sr(II), in turn, the dose of about 30 g/L is the optimum.

In turn, the AMF process used for water purification from Co(II) and Am(III) radionuclides appears efficient even for the smallest doses of sorbent. For Cs(I) and Sr(II) removal, the dose must be at least 7.5 g/L. It has also been found that several minutes of phase contact time is sufficient for carrying out the process.

Adsorption can be combined with several other water treatments, e.g., membrane filtration. Such a hybrid process is believed to be a potent replacement for the column process popularly used on an industrial scale. So, we have compared a hybrid adsorption-UF purification strategy of aqueous solutions with the one-step adsorption process. The obtained results indicated that the removal efficiency (E_M_) in the hybrid system based on using the FA is not significantly greater than this for the adsorption. Nevertheless, a hybrid system used on an industrial scale appears to have greater potential both than adsorption (due to the ease of removal of the loaded sorbent, concentrate, and retentate) and the column process (due to the relatively high speed of the purification process, and the relatively small size of the installation).

However, in the case of liquid radioactive wastes containing non-radioactive metal nitrates and chlorides, this sorbent has limited applicability. In such a key, it may be proposed to dilute the contaminated water prior to starting its purification.

## Figures and Tables

**Table 1:** Basic properties and chemical composition of the fly ash tested as the potential sorbent.

Parameter		Value	
Moisture		1.1%	
Grain size (diameter)		0.20–0.10 mm (41.2%) 0.10–0.05 mm (48.2%) 0.05–0.03 mm (8.6%)	
Bulk density:		1.79 g/mL	
Mass loss below 650 °C		15%	
Mass loss above 650 °C		2%	
ζ-potential (pH 5)		−21.6 mV	
ζ-potential (pH 8)		−24.8 mV	
Element	Element concentration in %, based on actual atomic mass (EDX) *	Constituent	wt. (%)
Oxygen	42.90 ± 0.80		
Carbon	26.49 ± 1.65		26.5
Silicon	12.08 ± 1.76	SiO_2_	25.9
Aluminium	9.12 ± 1.21	Al_2_O_3_	17.2
Calcium	3.42 ± 0.41	CaO	4.8
Iron	3.05 ± 0.18	Fe_2_O_3_	8.7
Potassium	1.27 ± 0.20	K_2_O	3.1
Sulfur	0.76 ± 0.17	SO_2_	1.5
Titanium	0.64 ± 0.04	TiO_2_	1.1
Magnesium	0.26 ± 0.10	MgO	0.4
Other **			10.8

* Measured for four surface areas; ** Oxides, mainly of Ba, Cu, Cr, Mn, Zn, Ni (as measured by the ICP-MS).

## Data Availability

Not applicable.
